# Robot-assisted vesico-vaginal fistula repair: technical nuances

**DOI:** 10.1590/S1677-5538.IBJU.2020.0749

**Published:** 2020-12-20

**Authors:** Alessandro Antonelli, Alessandro Veccia, Tonino Morena, Maria Furlan, Angelo Peroni, Claudio Simeone

**Affiliations:** 1 University of Verona Urology Unit AUOI Verona Urology Unit Verona Italy Urology Unit, Urology Unit AUOI Verona, University of Verona, Verona, Italy; 2 University of Brescia Urology Unit ASST Spedali Civili Hospital Brescia Italy Urology Unit ASST Spedali Civili Hospital, University of Brescia, Brescia, Italy

## Abstract

**Introduction:**

Vesico-vaginal fistula (VVF) is a rare event in Western countries and are mainly consequent to iatrogenic injuries (1, 2). When conservative management fails, surgical repair is needed, although timing and surgical approach (open or minimally invasive (3)) are still controversial (4, 5). Herein we present a step-by-step description of robot-assisted vesico-vaginal fistula repair.

**Material and Methods:**

From 2015 to 2018 six patients underwent robotic vesico-vaginal fistula repair.

Pre-operative cystoscopy was performed to identify the fistulous tract. The ureters were stented. A small catheter was inserted in the fistula. A longitudinal cystotomy was performed, then a dissection of the posterior bladder from the anterior vaginal wall was performed and the fistolous tract was excised. The vagina was sutured horizontally. Four patients underwent omental flap and two pericolic fat interposition. The bladder was closed with a double-layer suture.

**Results:**

All the vesico-vaginal fistulas developed after previous gynaecological surgery. The median operative time was 160 minutes [interquartile range (IQR) (146-177)]. Intraoperative blood loss was 25 (IQR 0-50) mL. No post-operative complications were recorded. Ureteral stents were removed at 4th post-operative day. Catheter was removed 13 (IQR 11-15) days after surgery after cystography assessment. One patient had Clavien I complication (ileus). Surgical pathology report was negative. No fistula recurrence was reported during follow-up.

**Conclusions:**

In our experience, robot-assisted fistula repair is a feasible and safe procedure. It presents the advantages of minimally invasive approaches and seems to provide low morbidity and good outcomes. Compared to transvaginal approach, the robotics allows to manage more complex cases with high success rate (6).
